# Associations between perceived discrimination and health: a cross-sectional public health study

**DOI:** 10.1186/s12889-025-23888-6

**Published:** 2025-08-01

**Authors:** Jan Georg Friesinger, Siri Håvås Haugland, Dina von Heimburg, Ottar Ness, John-Kåre Vederhus

**Affiliations:** 1 Department of Psychosocial Health, University of Agder, Grimstad, Norway; 2 Department of Education and Lifelong Learning, Norwegian University of Science and Technology, Trondheim, Norway; 3 Addiction Unit, Sørlandet Hospital, Kristiansand, Norway

**Keywords:** Discrimination, Health, Mental distress, Sociodemographics, Multiple disadvantages, Intersectionality

## Abstract

**Background:**

Many people experience discrimination based on factors such as age, gender, illness, disability, ethnicity, skin color, religion, beliefs or sexuality. Discrimination can lead to marginalization and be linked to poor health. As a result, there is a growing interest in exploring the underlying causes of discrimination and its cumulative consequences. The aim of the present study was to explore the prevalence of perceived discrimination (PD) in a large cross-sectional public health study and its association with self-rated health and mental distress.

**Methods:**

The study was based on data from 18,517 participants in the 2023 Norwegian Counties Public Health Survey (NCPHS) in Agder County. The participants were asked if they had experienced discrimination in the past 12 months and could specify different reasons. Furthermore, they were asked about their self-rated health, mental distress, and sociodemographic factors. In the analysis of the responses, PD was categorized based on the number of reasons, ranging from no PD to one, two, or three or more reasons.

**Results:**

Among the participants, 17.8% reported experiencing discrimination for at least one reason. Compared with those who experienced no discrimination, participants reporting discrimination for one reason were twice as likely to report poor self-rated health or mental distress. The odds increased to a range between two and a half to three times for those facing discrimination on two grounds. For individuals experiencing discrimination for three or more reasons, the likelihood rose substantially: Four times higher for poor self-rated health and approximately three times higher for mental distress.

**Conclusions:**

There is a clear association between multiple reasons for PD and low scores in mental and/or physical health, suggesting a cumulative influence. To address this issue effectively, prevention efforts should be implemented at both the national level and within local public health strategies.

## Background

Discrimination, characterized by the unfair treatment and judgment of individuals, is prevalent across countries. It has severe consequences ranging from structural to interpersonal levels and significantly impacts individuals’ health and well-being [1, 2]. While interpersonal discrimination refers to encounters where individuals engage in discriminatory actions toward others, structural discrimination refers to unfair treatment that is maintained by institutions or policies [3–5]. The negative judgment and mistreatment of individuals and groups can be based on characteristics such as age, gender, skin color or ethnicity, religion or belief, sexuality, disability or illness. These factors often intersect, leading to multiple and complex disadvantages [6–8]. People encounter discrimination in daily life, which arises in various settings, such as workplaces, schools, hiring processes, housing, healthcare, law enforcement interactions, and public spaces [9, 10]. Intersectionality refers to multiple forms of inequality or disadvantage that tend to compound themselves and create obstacles that are often overlooked or not well understood among conventional ways of thinking [11]. For example, a disabled woman who is part of an ethnic minority and is seeking daily assistance encounters severe problems in getting the help she needs due to her intersectional identity. Thus, intersectionality can contribute to increased discrimination in more subtle ways [12]. In addition to its negative impact on health, discrimination also leads to social disadvantages, both of which underscore the importance of studying it from a public health perspective [9, 13].

Being aware of the adverse societal effects of discrimination, policymakers have established protective measures for human rights and enacted national laws against discrimination [14]. However, substantial discrimination still persists among different groups [1, 2, 15, 16]. Racism, for example, is a severe issue that operates on different levels and is rooted in a hierarchical ideology, resulting in the classification of social groups based on ethnicity/skin color and their subsequent marginalization [17]. This ideology reflects a worldview in which a dominant group (e.g., white individuals) is perceived as superior to other groups (e.g., black and other non-white populations), who are viewed as inferior [18]. The consequences of racism include societal inequities and adverse health outcomes, such as poorer physical and mental health [13, 19]. While racism has been extensively studied in the U.S [20]., there is also evidence in Nordic countries, including Norway, that individuals with a history of migration face discrimination when applying for jobs [21, 22]. Another study revealed that immigrants in Norway who perceived discrimination (PD) were at a greater risk of experiencing mental health problems [23].

Discrimination is rarely a singular phenomenon, as it can manifest in multiple forms throughout an individual’s life course [24]. This is because individuals often experience discrimination for different reasons and in various situations. An acknowledged model that helps in understanding the effects of discrimination on people’s health is Krieger’s [3] ‘ecosocial theory’, which accounts for the life course and environmental levels. With this epidemiological lens, racism produces ethnic health inequalities that manifest as health inequities through specific pathways [3, 9]. The review and meta-analysis by Williams et al. [13] support the evidence that racism is a risk factor for disease and contributes to health disparities. In a longitudinal study, the association between age discrimination and an increased risk of severe health problems was highlighted, particularly with respect to whether participants had experienced discriminatory situations more frequently [2]. Several reviews have shown that PD can influence people’s mental and physical health both directly and indirectly [4, 25, 26]. Directly, discrimination itself (e.g., being treated unfairly) can cause psychological or physical harm, such as immediate stress, anxiety, or physical health impacts such as high blood pressure. Indirectly, it can affect health through coping mechanisms, such as engaging in unhealthy behaviors (e.g., smoking or overeating), which further impact well-being. Over time, discrimination can also contribute to allostatic load—a cumulative physiological burden imposed on the body through prolonged exposure to stress [7, 27].

The literature advocates for an association between discrimination and health, indicating that multiple forms of discrimination together can exacerbate the negative health effects of discrimination and account for more than one type of discrimination alone [9, 28]. A critical review of studies has confirmed evidence that multiple types of discrimination negatively affect mental health [29]. However, the review noted that existing studies often rely on small and non-representative samples. Our study seeks to advance this body of research by using a large, population-based sample with random sampling, thereby improving generalizability [30]. Furthermore, we contribute to the field by examining PD in a Scandinavian context—a region often regarded as culturally egalitarian [21, 31]. Investigating the prevalence and consequences of PD in such settings is important, as it challenges assumptions of equality and adds nuance to cross-cultural discrimination research. How multiple forms of discrimination affect people’s health can be explained through intersectional studies [12, 24, 32–34]. Intersectionality is a concept highlighting the diverse experiences individuals face based on their social identities, including factors such as ethnicity, class, gender, and sexuality. However, while discrimination often intersects, more research is needed to understand how cumulative discriminatory experiences affect health outcomes. To some extent, this cumulative effect may contribute to the allostatic load.

### Objectives

The present study aimed to examine the prevalence of perceived discrimination (PD) in a large population-based health survey in Norway and its association with poor self-rated health and mental health distress.

## Methods

### Participants and data collection

The Norwegian Counties Public Health Survey (NCPHS) was conducted in 2023 in Agder County [30, 35], collecting cross-sectional data from individuals aged 18 years or older regarding their health, well-being, injuries, health behaviors, and environmental factors. A total of 57,891 individuals in Agder were selected through a random sampling method utilizing the Norwegian Population Registry, which includes information about residents in Southern Norway.

To contact the participants, their email addresses and telephone numbers were obtained from the contact registry maintained by the Agency for Public Management and eGovernment (Difi). The survey was carried out online by Kantar Public (now known as Verian) on behalf of the Norwegian Institute of Public Health and Agder County Municipality. To ensure data accuracy, participants were required to provide their age and gender, which were cross-checked against the Population Registry. Before they participated in the survey, the individuals provided electronic consent. To protect privacy, personal identification numbers were removed from the collected data before being transferred to the researchers. Data collection occurred from September 20th to October 23rd, 2023, with 18,517 inhabitants of Agder participating, resulting in a response rate of 32.0% [35]. Despite the representativeness of the sample, some groups showed low participation, such as young adults (< 30 years), the oldest women (80 + years) and participants with lower education levels.

### Measures

Demographic information such as age and gender (female/male) was retrieved from registries. In this study, age was available as a continuous variable, with individuals older than 84 years coded as 85 for anonymity reasons (*n* = 114).

#### Self-rated health

Self-rated health (SRH) was measured with the SRH-5, a commonly used epidemiological measure that captures individuals’ subjective perceptions of their health status. It is regarded as a valuable tool for predicting significant outcomes, including mortality [36, 37], given in a cultural context [38]. The participants were asked to rate their general health status using the following ordinal scale: (1) very good, (2) good, (3) neither good nor poor, (4) poor, and (5) very poor. In line with previous studies that used the SRH-5 [39, 40], we dichotomized the SRH-5 scores into two categories: ‘good self-rated health’ (responses 1–3) and ‘poor self-rated health’ (responses 4–5).

#### Mental distress

A shortened version of the Hopkins Symptom Checklist, known as the SCL-5, was utilized to assess mental distress. The participants were asked to indicate whether they had felt sad or depressed, hopeless about the future, tense or anxious, constantly fearful, or worried in the past week. Responses were scored on a 4-point ordinal scale ranging from “not bothered” to “extremely bothered”. The scores were averaged, and the scale was dichotomized on the basis of the recommended cut-off at 2.0, as suggested by Strand et al. [41]. Scores above 2.0 were considered indicative of clinically significant mental distress, whereas scores of 2.0 or below were interpreted as non-clinical values. The SCL-5 has proven to be as effective as longer versions such as the SCL-10 and SCL-25 and has high reliability, with a Cronbach’s alpha of 0.87, according to a validation study conducted in Norway [41].

#### Perceived discrimination (PD)

The measures of PD were inspired by scales used in other studies [29]. PD was retrieved by asking participants if they had experienced being treated worse than others in the past 12 months due to ‘age’; ‘gender’; ‘health problems, illness, injury’; ‘disability’; ‘ethnic background’; ‘skin color’; ‘religion/belief’; ‘political views’; ‘sexual identity’; or ‘other reason’ with response options of “yes”, “no”, “don’t know”, or “refusal”. If all the responses were marked as “no”, it indicated that there was no PD (coded as 0, reference category), responses “don’t know” and “refusal” were treated as missing. We computed a variable indicating PD if respondents reported experiencing discrimination for at least one reason. Second, all the items indicating PD were summed. This summed variable was then categorized into four groups: no discrimination (0), discrimination for one reason (1), for two reasons (2), and for three or more reasons (3+).

#### Financial difficulties

The assessment of financial difficulties was self-reported through the following question: For individuals living alone, please consider your total income. If you live with others, think about the combined income of your household. How easy or difficult is it for you to manage your daily expenses with this income? We categorized responses of (1) very difficult, (2) difficult, and (3) somewhat difficult as indicating financial difficulties, whereas responses of (4) somewhat easy, (5) easy, and (6) very easy suggested no financial difficulties. Response option “don’t know” was coded as missing,

#### Social support

Social support was assessed with Oslo social support scale (OSSS-3) [41], asking “How many people are so close to you that you can count on them if you have great personal problems?” with response options: none, 1–2, 3–5, and 5 +. The next questions were scored on 5-point ordinal scales: “How much interest and concern do people show in what you do?” (response options: from ‘none’ to ‘a lot’), and “How easy is it to get practical help from neighbors if you should need it?” (response options: from ‘very difficult’ to ’very easy’). The sum score of three items was categorized into poor social support (3–8), moderate social support (9–11), and strong social support (12–14).

#### Education

The educational attainment level was reported according to the International Standard Classification of Education (ISCED) 1997. To ensure comparability across countries and correspondence with ISCED 2011, the levels were coded as recommended by Eurostat [42] into three aggregates: low (levels 0–2), medium (levels 3–4), and high (levels 5–6) education.

#### Migration background

Migration background was assessed with the following question: “Were you, or at least one of your parents, born outside of Norway?” Responses marked with “yes” indicated a migration background, whereas responses marked “no” suggested that the individual and their descendants were not foreign-born. Participants who responded with “yes” received a follow-up question: “Which country were you, your mother, and your father born in?” We coded the responses into Norwegian-born with two Norwegian-born parents (no migration background), Norwegian-born with either one or two foreign-born parents, or foreign-born [43]. Additionally, we grouped the countries into Global North (Europe, North America, Australia and New Zealand and South) and Global South (Asia, Africa and South America). Inconsistent and invalid responses were treated as missing data.

### Data analysis

We conducted descriptive analyses of the overall distribution of PD, including the prevalence of each reason for PD. Multivariable logistic regression was also conducted with self-rated health and mental distress as dependent variables. PD was included as the primary exposure variable, while sociodemographic variables were included as control variables in these models. The results are reported as adjusted odds ratios (ORs) with 95% confidence intervals (CIs), and the level of statistical significance was set to 5%. All analyses were conducted using IBM SPSS Statistics 30.0.

## Results

The participants’ mean age was 51.5 years, with a standard deviation of 16.5 years. Table 1 shows the sample characteristics, whereby the majority of participants were female (55.7%), had no financial difficulties (73.8%), and had no migration background (83.6%). Participants with migration background (16.4%) responded moreover as Norwegian-born with either one (21.1%) or two foreign-born parents (2.3%) or foreign-born persons (60.1%). The follow-up question had some missing values (16.5%) compared to the first question on migration background. Of the foreign-born persons (*N* = 1,820), 72.6% originated from the Global North, while 27.4% were from the Global South. Less than half (43.3%) of the participants had a high level of educational background, and one in seven (14.7%) reported poor social support. Almost one in four participants reported mental distress, and one in ten reported poor self-rated health. When reporting experiences of discrimination, 9.1% of the participants reported being discriminated for one reason, 4.9% reported two reasons, and 3.8% reported three or more reasons.

**Table 1:** Sample characteristics of the participants (*N* = 18,517)

	*N* (%)
Gender, female	10,316 (55.7%)
Age, mean (SD)	51.5 (16.5)
Education (*N* = 18,395)	
Low	6,922 (37.6%)
Medium	3,500 (19.0%)
High	7,973 (43.3%)
Financial difficulties (*N* = 18,074)	4,739 (26.2%)
Migration background (*N* = 18,417)	3,026 (16.4%)
Social support (*N* = 18,177)	
Poor	2,675 (14.7%)
Moderate	8,395 (46.2%)
Strong	7,107 (39.1%)
Mental distress (*N* = 18,471)	4,488 (24.3%)
Poor self-rated health (*N* = 18,451)	1,820 (9.9%)
Number of perceived reasons for discrimination (*N* = 18,411)	
No discrimination	15,129 (82.2%)
Only one reason of discrimination	1,681 (9.1%)
Two reasons of discrimination	901 (4.9%)
Three or more reasons of discrimination	700 (3.8%)

In total, 17.8% (*N* = 3,282) of the participants reported experiencing discrimination for at least one reason. To allow for multiple responses regarding discriminatory reasons in the survey, Table 2 illustrates a breakdown of these responses among participants who reported at least one PD (subset) and among the sample. The most frequently selected reason was ‘health problems, illness, injury,’ accounting for 37.5% of the responses within the subset (representing 6.7% of the sample), followed by gender at 31.9% (representing 5.7% of the sample).

**Table 2:** Perceived discrimination (PD) in the past 12 months for different reasons—multiple responses

Discrimination based on	Responses	% of respondents with at least one PD (*N* = 3,282)	% of respondents (*N* = 18,411)
	*N*		
Age	772	23.5%	4.2%
Gender	1,047	31.9%	5.7%
Health problems, illness, injury	1,230	37.5%	6.7%
Disability	595	18.1%	3.2%
Ethnic background	409	12.5%	2.2%
Skin color	184	5.6%	1.0%
Religion/belief	425	12.9%	2.3%
Political views	611	18.6%	3.3%
Sexual identity	160	4.9%	0.9%
Other reason	710	21.6%	3.9%

Table 3 displays the results from the multivariable logistic regression adjusted for sociodemographic variables (financial difficulties, social support, education, age, gender, and migration background). Those who reported PD for any number of reasons had significantly greater odds of reporting poor self-rated health or mental distress compared to the group who reported no PD. For example, while reporting PD for one reason doubled the chances of reporting poor self-rated health or mental distress, three or more reasons for PD increased the chances considerably for poor SRH (OR 4.30, 95% CI = 3.55—5.21) and mental distress (OR 3.17, 95% CI = 2.63—3.81). Among the control variables, reported financial difficulties, moderate or poor social support and a low level of education were positively associated with both poor SRH and mental distress, whereas gender (male) was negatively associated with these outcomes. A migration background was associated with mental distress. The explained variance (Nagelkerke R²) in poor SRH was 14.6%, and in mental health distress 28.9%. Regarding the model fit, the AUC (Area Under the ROC Curve) for predicting poor SRH was 0.74, and for predicting mental health distress was 0.79. These values can be considered as good model fits [44], particularly for mental health distress.

**Table 3:** Multivariable logistic regression of associations between perceived discrimination (PD) and poor self-rated health or mental health distress adjusted for sociodemographic variables (financial difficulties, social support, education, age, gender, and migration background)

	Poor self-rated health	Mental health distress
No perceived discrimination	ref	ref
One reason for PD	1.95 (1.66—2.28)***	1.97 (1.74—2.22)***
Two reasons of PD	3.02 (2.51—3.63)***	2.48 (2.12—2.90)***
Three or more reasons for PD	4.30 (3.55—5.21)***	3.17 (2.63—3.81)***
Financial difficulties (ref: no)	2.24 (1.99—2.51)***	2.34 (2.15—2.55)***
Social support, strong	ref	ref
Social support, moderate	1.61 (1.41—1.84)***	2.30 (2.09—2.52)***
Social support, poor	3.29 (2.83—3.83)***	5.55 (4.93—6.25)***
Education, high	ref	ref
Education, medium	1.21 (1.04—1.40)*	1.13 (1.01—1.26)*
Education, low	1.52 (1.32—1.72)**	1.28 (1.18—1.40)***
Age	1.01 (1,01—1,02)***	0.97 (0.97—0.97)***
Gender (ref: female)	0.82 (0.73—0.91)***	0.71 (0.66—0.77)***
Migration background (ref: no)	0.81 (0.70—0.94)**	1.12 (1.01—1.24)*

Data are presented as odds ratios (ORs) with 95% confidence intervals (CIs)

* *p* < 0.05, ** *p* < 0.01, *** *p* < 0.001

## Discussion

In this study, we investigated the associations between perceived discriminatory reasons and both poor self-rated health and mental distress using data from a population-based health survey. Our findings confirmed that the odds of rating one’s own health as poor or reporting mental distress significantly increase when more discriminatory reasons are perceived, suggesting a cumulative effect.

The prevalence of PD for various reasons in the whole population ranged from 0.9% (sexual identity) to 6.7% (health problems, illness, injury). Such figures, drawn from representative population studies, provide a basis for comparing the scope of discrimination across societies. For example, in the Eurobarometer survey conducted across EU countries, 1 in 5 respondents reported experiencing discrimination for at least one reason, which was quite similar to our finding (17.8%). Among the reported reasons, sexual orientation was the least common at 1%, whereas age was the most common at 6% [15]. Thus, our findings are comparable to the prevalence of discrimination observed in other European countries, providing an international context for our results. Although some categories, such as sexual identity and skin color, represent relatively low percentages (0.9% and 1%, respectively), these numbers should not be dismissed as minor issues. Given that the sample in this study is fairly representative of the general Norwegian population [35], even 0.9% of Norwegian adults represent a significant number of individuals—each with their own unique experiences and potential harms [4, 9, 13]. The impact of these discriminatory experiences can be deeply personal, especially for reasons that individuals may find it difficult to live openly with. These experiences can also involve external, visible characteristics—such as age, gender, skin color, or disability—that make individuals more easily identifiable as “standing out” in a social context, as pointed out in studies about racism [18, 45]. As mentioned, such experiences can have lasting negative consequences for mental health, social well-being, and overall quality of life.

Another factor that can exacerbate these negative consequences is living in a rural context. In 2024, approximately one-third of Agder’s population resided in sparsely populated areas, where “everyone knows everyone” and where it is more difficult to conceal traits that set one apart. In smaller communities, individuals may feel more vulnerable due to the lack of anonymity that larger cities typically offer. Moreover, rural areas have more often limited access to mental health services, support groups, and organizations that address discrimination. Adherence to traditional norms and lower tolerance for diversity may also contribute to more frequent or intense discrimination. Following Krieger’s life course approach, we assume that strong social support can serve as a protective buffer against experiencing poor self-rated health and mental distress [3]. Notably, the ORs for moderate or poor social support in relation to health outcomes were similar to those for PD. This suggests that strong social support may counteract the negative effects of PD [46], which appears to be the case in our study as well. Overall, we want to reiterate that the scale of the problem should not be trivialized. The possible harmful effects of PD were evident in the results of our logistic regression analysis. Experiencing discrimination for three or more reasons was associated with three times higher odds of reporting mental distress and four times higher odds of reporting poor health. This finding is significant, as previous research has confirmed that poor self-rated health is a predictor of mortality [37].

Intersectionality elucidates the interplay of social identities, which together can create unique challenges and forms of marginalization. An analysis limited to discrete identities or the summation of discriminating experiences, as employed in this study, is insufficient to capture the complex realities individuals face. However, we contend that measuring cumulative exposure can, to some extent, represent and contribute to the allostatic load, as was evident in our regression analyses. Future research should investigate whether specific clusters or overlapping patterns present distinct challenges and explore more nuanced ways in which intersecting identities shape experiences of discrimination [4, 47–49]. Additionally, the impact of interconnected experiences could be explored through qualitative research to gain a deeper understanding of the personal stories behind these experiences.

Our results indicate strong evidence of discrimination with adverse health effects in the Southern of Norway, despite government efforts to prevent discrimination and promote equity. This situation is comparable to the international status quo, where discrimination is recognized as a severe societal problem with disruptive consequences for individuals and their health, highlighting the need for interventions to reduce discrimination [1]. As such, we want to discuss public health interventions to end discrimination. It starts with the fact that discrimination needs to be seen as a multifaceted phenomenon where people often face more than one discriminatory reason.

Addressing these inequities necessitates intersectoral, system-wide approaches that consider overlapping social determinants of health through equity-focused policy tools at all government levels. At the state level, coherent legislation and social protection programs are crucial for tackling discrimination and promoting social inclusion. Institutional partnerships and measures across education, labor, and housing are key to ensuring fair employment and good living standards for all [31, 50]. At the local level, public services must be socially inclusive, culturally sensitive, and tailored to various social identities. Inclusion in local communities through neighborhoods, education, leisure activities, and organizational life is vital for preventing and addressing discrimination and its consequences [51, 52]. Both nationally and locally, it is essential to enhance antidiscrimination literacy and promote a human rights-based approach to health and wellbeing. Such an approach should focus on structural and relational recognition, nurturing mutual empathy and solidarity between population groups in everyday life settings. Moreover, it is vital to deepen democratic participation in co-creation of public values, where those most affected have real opportunities to impact the conditions, processes, and decisions that ultimately affect their lives [51, 53, 54].

## Strengths and limitations

A notable strength of this study is the large number of participants across a broad age range, drawn randomly from the general population. Drawing these samples from the Norwegian Population Registry, an updated and reliable official source, can be considered a strength of the study. However, we acknowledge that only individuals registered as ‘ordinary’ residents at the time of data collection were eligible for inclusion. This criterion may have excluded recent movers to Agder County and newly arrived immigrants to Norway. Additionally, our sample included a higher proportion of adults with higher education compared to the overall adult population in Norway. The relatively low response rate of 32.0% compared to the previous NCPHS Agder 2019 (45.5%) poses a risk of non-response bias. This decline in participation rates appears to be a broader trend across all NCPHS surveys following the COVID-19 pandemic [30].

Low response rates raise the concern that those who choose to participate may systematically differ from non-responders in their opinions, experiences, health behaviors, or other characteristics relevant to the survey topic. Moreover, lower response rates among elderly women and young men might lead to underrepresentation. This skew may have led to an underestimation of the prevalence of PD. Exposure to discrimination was assessed using retrospective questions about experiences in the past 12 months. This approach is vulnerable to recall bias and loss of contextual details, which can complicate accurate assessment. More obvious acts of discrimination are typically more memorable than subtle ones, but subtle forms can accumulate significant impact over time, potentially being overlooked in individual instances. Thus, retrospective assessments often disproportionately capture more overt experiences.

The cross-sectional nature of the study moreover limits the ability to infer causality from the observed associations. Consequently, although the findings provide valuable insights into potential relationships, they should be interpreted with caution. While the question regarding migration background had almost no missing values, the follow-up question about the birth country showed a higher proportion of missing values. Therefore, we used the overall migration background variable in the regression analyses, while the birth country variables were used only to provide a more detailed description of the sample. Even though SRH is an acknowledge self-reported health measure [36–40], dichotomizing the SRH may lead to some loss of information. Our survey was informed by well-established scales, including the Experiences of Discrimination Scale [10] and the Everyday Discrimination Scale [45]. While these scales are more extensive and incorporate questions about the frequency of discrimination, we were unable to incorporate them in full, resulting in the loss of some detail from the original measures. We acknowledge that the cumulation of PD reasons might obscure the intersectional nature of PD and the relative impact of different types of PD. Nevertheless, our survey captures key aspects of perceived discrimination, aligning with approaches used in similar large-scale studies [29].

## Conclusion

The influence of PD on mental distress and self-rated health was significant, showing that cumulative PD was associated with worse outcomes. Therefore, efforts to prevent discrimination should be a goal of both national and local public health initiatives.

## Data Availability

The data were provided by the Norwegian Institute of Public Health, with permission. The dataset supporting the conclusions of this article is available in the repository Helsedata (https://helsedata.no/en/) upon application.

## Abbreviations

AUC: Area under the ROC curve
CI: Confidence intervals
Difi: Agency for Public Management and eGovernment
ISCED: International Standard Classification of Education
NCPHS: Norwegian Counties Public Health Surveys
OR: Odds ratio
OSSS-3: Oslo social support scale
PD: Perceived discrimination
QoL: Quality of life
SCL-5: Mental distress - a shortened version of the Hopkins symptom checklist
SD: Standard deviation
SRH: Self-rated health
